# Get vaccinated or else…employees’ perspective on mandatory vaccination in the retail sector in Zimbabwe

**DOI:** 10.3389/fpsyg.2022.946454

**Published:** 2022-11-18

**Authors:** Martha Mapuranga, Farai Maunganidze, Shaun Ruggunan

**Affiliations:** School of Management, IT and Governance, College of Law and Management, University of KwaZulu Natal, Durban, South Africa

**Keywords:** COVID-19, vaccination, employee, retail sector, perspective

## Abstract

The emergence of COVID-19 has resulted in many changes in the world of work. Measures such as remote working, physical distancing, compulsory use of face masks, sanitization among others. With time, a number of medical interventions to deal with the pandemic were developed and availed. Zimbabwe’s retail sector was not spared of different vaccines which were meant to curb the virus. Most Zimbabwean organizations made it mandatory for their employees to get vaccinated or risked losing employment. However, less is known about the perceptions of employees toward voluntary vaccination. This gap is important given the strategic nature of employees in an organization. This paper poses the following questions (1) to what extent were employees consulted on the compulsory vaccination? (2) What are the employees’ perceptions toward compulsory vaccination? (3) How are employees coping with the mandatory vaccination? The study was premised on the classical work of Kurt Lewin on types of leadership, specifically autocratic-democratic styles. Twenty shopfloor employees from two major retail outlets with functional human resource departments and works councils in Masvingo were purposively sampled and interviewed using a semi-structured interview guide. The sample composed of women and men of different age groups. Thematic analysis was used to analyze data. The paper argues that employees have a right to be involved in issues that concern them. The study has established four levels of consultation existing on a continuum namely formal and genuine consultations, formal but less genuine consultations, informal consultations, and no consultation at all. The fourth level emerged to have been the most popular among most participants. With regards to employees’ perceptions of mandatory vaccination by management, findings have revealed three categories which are, perceived good decision, perceived tight hands on the part of management and the them and us perceptions. Concerning reactions to mandatory vaccination, the study has shown that employees in the retail sector had a number of options to follow. Some went for full vaccination willingly or under duress, while others settled for a single dose. Most participants highlighted that they fraudulently obtained some vaccination cards. These findings support the relevance of engaging employees on matters that affect them. The study has therefore established the importance of genuine consultations between management and employees on issues that pertains the latter.

## Introduction and background

Toward the end of 2019, the world woke up to the emergence of COVID-19, also known as corona virus. The pandemic was first discovered in Wuang, China (Yang et al., 2020) and quickly spread to the rest of the world (Sultana et al., 2020). COVID-19 is caused by the recently discovered severe acute respiratory syndrome coronavirus (SARS-CoV-2). Due to its devastating effects, Watkins (2020) describes the pandemic as a global threat. As of 3 July 6.3 million people had succumbed to COVID-19, with an average of 4.6 million new cases being reported (WHO, 2022). It is these alarming figures that forced governments, with the assistance of the World Health Organization (WHO) Guidelines to come up with measures meant to reduce the spread of the pandemic.

A raft of measures including promoting physical/social distancing as well as persuading the population to follow health behavioral guidelines (Nofal et al., 2020) were outlined. Zimbabwe was not spared from the efforts of controlling the spread of the pandemic (Chigevenga, 2020). Of interest in this study was the “voluntary” COVID-19 vaccination. In several countries, the vaccination was met with mixed reactions. On one hand, some people saw it as a positive move meant to save lives (Coe et al., 2022) while on the other side, some saw it as risk as they argue that this was an opportunity by some people to wipe away some sections of the population (Qiao et al., 2020).

In Zimbabwe, although the vaccination was voluntary, most organizations, including the government started subtly forcing their workforce to get vaccinated. Unvaccinated employees were told not to report for duty until they are vaccinated (Aaron and Tafadzwa, 2021; Matikiti, 2021). Failure to report to work meant no remuneration and eventually would lead to loss of employment. Most employees saw the move by their respective employers as abusing their freedom to choose as they had no choice but to get vaccinated so as to save their jobs (Kugarakuripi and Ndoma, 2022), especially considering the high rate of unemployment which has characterized Zimbabwe in recent years. Government and other business experts argued that they wanted to protect their vaccinated employees, customers and other stakeholders against unvaccinated employees.

It is against this background that the study seeks to establish the perceptions of retail employees with regards to voluntary vaccinations which has been interpreted in some circles as mandatory. Less is known about the perceptions of these employees, especially considering the precarity of work in the retail industry. In addressing the gap in literature, the paper was guided by 3 questions namely (1) to what extent were employees consulted on the compulsory vaccination? (2) What are the employees’ perceptions toward compulsory vaccination? (3) How are employees coping with the mandatory vaccination?

Previous studies on COVID-19 in the workplace have focused on implications, issues, and insights for future research and action (Kniffin et al., 2021), making a workplace ready for COVID-19 (WHO, 2020), workplace COVID-19 vaccination (Riva et al., 2022) and COVID-19 related mental health effects in the workplace (Giorgi et al., 2020). This study took a different perspective by focusing on the perceptions of employees of mandatory COVID-19 vaccinations, particularly in a precarious environment such as the one obtaining in Zimbabwe.

The paper will start by focusing on the concept of autocratic-democratic leadership styles before proceeding to a brief discussion on the economic trajectory traveled by Zimbabwe from independence. Thereafter attention is turned to COVID-19 in the workplace before attending to issues pertaining to dynamics and debates around COVID-19 vaccinations. The paper proceeds to look into aspects involving employee involvement and participation. Methodology used in the paper is discussed Barthold et al. (2022) before the presentation of findings and discussion. The paper continues to attend to recommendations before it concludes with a conclusion section.

### Democratic leadership

The study is premised on the work of Lewin (1944) on democratic leadership, which is also known as participative leadership or shared leadership. This is a leadership style that entails members of a group participating in the decision-making process. In participative leadership, there is collective decision-making between managers and subordinates.

In the context of this study, democratic leadership meant retail managers creating an environment where employees would formally and freely participate on issues to do with COVID-19 vaccination. As noted by Kilicoglu (2018), through democratic leadership, a sense of ownership is developed with the participation of all members of an organization. Regarding COVID-19 vaccination, myths and misunderstandings surrounding the issue would be addressed and dispelled, leaving employees prepared to go through the process without a sense of being coerced.

On the opposite of the spectrum lies autocratic or authoritarian leadership. This often comprise of leaders in possession of ultimate authority and power over others. These leaders make choices based upon their ideas alone and do not listen to their team members or seek input from others. Autocratic leadership has gained through such aspects as punishment, threat, rules and regulations and demands (Chu, 2014; Erdem, 2021).

However, with caution, autocratic leadership style works in some situations. Researchers concur that in times of a crisis, some autocratic traits of leadership style must be practiced (Du Plessis and Keyter, 2020; Ma and Yang, 2020). When dealing with a crisis, leaders are expected to make difficult decisions, communicate and execute a strategy with an unwavering focus.

By its very nature, COVID-19 was an issue requiring urgent attention in organizations (Hodder, 2020). There was need to quickly come up with ways that would save the organizations, its operations and stakeholders and Zimbabwe was not spared of this need. From an autocratic leadership perspective and within the context of COVID-19 in Zimbabwe, managers had to make quick decisions to protect both the organizations and stakeholders.

We submit to the notion that COVID-19 required an urgent approach to decision making and at the same time, employees also needed to be taken on board in one way or the other. There was therefore a need to balance an autocratic leadership style with the democratic leadership style. It was also important to keep formal communication channels between managers and employees open, especially pertaining COVID-19 vaccines and the vaccination process.

### Employee involvement and decision making

Employee involvement entails direct participation of staff meant to help an organization fulfill its mission as well as its objectives by applying their own ideas, efforts and expertise toward solving problems and making decisions. It has been argued that employee involvement makes employees feel part of the family. The result is that employees become more responsible about their work. Such an environment cultivates possibilities of innovative thinking and ideas to address challenges in the workplace (Bratton et al., 2021).

Job satisfaction increases when employees are involved in decision making (García et al., 2018). Similarly, Dahmardeh and Nastiezaie (2019) has it that employees are bound to be committed to decisions they would have participated in. Employees feel honored and valued when they are consulted and the other way round is true (García et al., 2018).

There is actually more need for organizations to engage their employees during times of crisis. Times of crisis call for organizations to continuously engage their workers in decision making processes. This has been argued to harness a feeling of commitment and a high level of motivation required by both parties during a period of a pandemic (Chanana, 2021).

However, Hodgkinson (2018) opines that a sense of managerial prerogative in making decisions may be seen in efforts by management to avoid involving unions and employees when it comes to crucial decisions. Kougiannou et al. (2021) echo similar views when they argue that management’s perception of risk about sharing and discussing information with employees and employee representatives influences their decision whether to involve employees or their unions in decision making processes in organizations. In the case of a pandemic and its devastating effects, like that involving COVID-19, management may see it un-worthwhile, risk and time consuming to involve employees and may proceed to make unilateral decisions

### Zimbabwe’s economic trajectory and employment

Zimbabwe got its independence from Britian in 1980. The first few years after independence were characterized by steady growth (Mashizha and Mapuva, 2018; Musavengane, 2018). The economy started showing signs of distress. Economic decline was precipitated by a myriad of events including payment of gratuities to veterans of the liberation war (Mazorodze, 2020), the introduction of the Movement for Democratic Change as a new and powerful opposition party (Hadebe, 2019), land reform (Mkodzongi and Spiegel, 2019), corruption (Muzurura, 2019), and economic sanctions (Mazorodze, 2021), among other factors.

An unstable economic environment in Zimbabwe has led to company closures (Gukurume, 2018), downsizing (Chirasha and Sauti, 2020), and depressed foreign direct investment (Bonga, 2020). These developments in organizations have worsened an already battered economy, thus making it more difficult for those in employment as well as those in search of employment.

### COVID-19 and the workplace

COVID-19 has reconfigured the workplace, not only in Zimbabwe but around the world (Kniffin et al., 2021). In most organizations, in line with their respective governments’ directives and World Health Organization COVID-19 regulations, had to change the way they have been operating (de Lucas Ancillo et al., 2021). Organizations had to start practicing social distancing (Noh et al., 2020), workplace sanitisers (Bhaumik, 2021), office decongestion (Balisi and Madisa, 2021). Of interest to this study, these measures were later followed by voluntary vaccination. Zimbabwe was not spared of COVID-19 mandatory vaccination in its quest to control its devastating effects (Murewanhema et al., 2022).

### COVID-19 vaccination

Some world pharmaceutical companies reacted to the pandemic by coming up with drugs which were meant to protect people from getting infected. The drugs included Sinopharm BIBP, Covaxin, Sinovac and Sputnic V and Zimbabwe adopted the use of all the 4 drugs. However, just like in many other countries, citizens had mixed perceptions regarding the vaccinations. As noted by McAbee et al. (2021), vaccines remain one of the most effective public health strategies meant to protect against infectious diseases yet vaccine hesitancy has emerged as a health threat globally. The same hesitancy has characterized the uptake of COVID-19 vaccination roll out in Zimbabwe (Murewanhema et al., 2022). Kugarakuripi and Ndoma (2022) acknowledges that lack of trust in government exacerbated by reliance on social media for facts have been instrumental in Zimbabweans resisting COVID-19 vaccinations. However, as noted by Mugari and Obioha (2021), there is always need for responsible authorities to emphasize the benefits of COVID-19 vaccination so that people voluntarily participate in such activities without a sense of being forced.

Although on paper, in Zimbabwe vaccination was said to be voluntary it was subtly made compulsory. Some people got vaccinated so as to access public spaces, some complied in line with directives from their employers while others complied to satisfy school requirements (Chigevenga, 2020; Makadzange et al., 2022).The government of Zimbabwe also introduced mandatory vaccination for its employees (Makadzange et al., 2022). Of interest to this study was the mandatory vaccination of employees in the retail sector in Zimbabwe. Frontline workers have not only been mandated for vaccination in Zimbabwe but it has been a global trend in other countries as well, for instance in United States, (Prince et al., 2022), in India, (Bagcchi, 2021), and in Italy, (Craxì et al., 2021).

Regarding mandatory vaccination in Zimbabwe Kugarakuripi and Ndoma (2022) have it that the Zimbabwe Congress Of Trade Unions (ZCTU) unsuccessfully tried stop the exercise. Employees were left with no other option besides being vaccinated. Most organizations compelled their employees to be vaccinated. Retail employees were not spared of this vaccination wave.

## Materials and methods

### Design

The study adopted a phenomenological approach which is a type of qualitative inquiry that emphasizes experimental, lived aspects of a particular construct. The focus is on how the phenomenon is experienced at the time of its occurrence (Coolican, 2018). In this context, retail employees had to express their lived experience regarding the mandatory COVID-19 vaccination in Zimbabwe.

### Participants and procedure

Braun and Clarke (2021) recommended a minimum sample size of at least twelve for quantitative studies, therefore twenty employees (males: *n* = 10, females: *n* = 10, age range 18–47 years) were purposively sampled from two major retail outlets in the town of Masvingo, Zimbabwe. Purposive sampling allows the researcher to select participants because of the defining characteristics that makes them holders of the data needed (Olurotimi, 2018). Retail employees was one of the categories of workers who continued attending to their duties at work even when other categories made such arrangements as working from home. The choice of two major retail outlets was reached on the realization that such organizations have full-fledged human resource departments as well as works councils representing employees. It was assumed that having a human resource management department in place meant the existence of a structure that can be used in addressing and consulting employees on issues that affect them. Furthermore, it was also assumed that the existence of a works council meant interests of employees were negotiated between the employer and the employees. Anonymity of participants was maintained and pseudonyms were used.

The inclusion criterion was that participants were retail shop employees who had worked for the respective shops in Masvingo, Zimbabwe branches for not less than 5 years as they have better experience of working in the retail sector. Researchers were assisted by the human resource officers of the respective retails shops to purposively sample the participants according to the inclusion criterion. Detailed information about the study, including all ethical related, issues was provided to all the participants prior to their participation. After explaining the research to prospective, participants, those who were willing were given consent forms to complete. Thereafter, confidentiality was explained to all participants. Participants were also told that they were free to withdraw from the interview anytime during the process without any repercussion. In addition, participants were also told that there were no right or wrong answers. Participants were also consulted before recording them. Gender and age were taken into consideration to ensure that the sample encompassed represented different categories of participants. Table 1 below depicts the sample characteristics.

**Table 1:** Sample biographical characteristics.

Retail outlet A		Retail outlet B	
Males	5	Males	5
Females	5	Females	5
**Age ranges (years)**		**Age ranges (years)**	
20 and below	2	<20	1
21–25	3	20–25	2
26–30	1	26–30	2
31–35	2	31–35	1
36–40	1	36–40	1
41+	1	41+	3

## Data collection

Interviews were conducted by MM and FM and SR analysed the data. MM is PhD student with the University of KwaZulu Natal, department of human resource management in South Africa. FM is a research associate with the University KwaZulu Natal, South Africa and a senior lecturer with the Great Zimbabwe University, department of human resource management. SR is a Professor at the University of KwaZulu Natal, South Africa.

Data collection was conducted between March and April 2022. Although in Zimbabwe, COVID-19 vaccination started in 2021, the move gained momentum at the end of 2021 and early 2022 when vaccines were made available to all adults aged 18 and above in Zimbabwe. Interviews were conducted in person with all World Health Organization COVID-19 protocols fully observed. The interviews had a duration of between 27 and 48 min. Each interview session was recorded transcribed. Data collection periods were arranged by the respective human resource officers during working hours at the employers’ premises. After each interview session, participants were thanked, and no rewards were provided for participating. Conducting such a study at the employer’s premises posed some risk to employees as the former could have made a conscious effort to overhear the interviews, possibly leading to the victimization of some employees. The researchers however took the word of the senior managers that the organization is a learning organization and wanted to learn on how best they can take care of the interests of their workforce. However, in an effort to make sure employees were safe, the interviews were conducted in the middle of the conference room, doors and windows were all closed to guarantee as much privacy as possible. The study is presented in accordance with the Consolidated Criteria for Reporting Qualitative Research (COREQ), (Tong et al., 2007).

The study made use of a semi-structured interview guide/schedule to collect data (Hamilton and Finley, 2019). The instrument composed of four sections namely;

- Demographic information
- The extent to which employees have been consulted regarding mandatory COVID-19 vaccinations,
- Perceptions of employees relating to mandatory COVID-19 vaccinations and,
- Ways through which employees are coping with mandatory COVID-19 vaccinations.

### Data analysis

Data was analyzed using thematic analysis (Braun and Clarke, 2021). The analysis took an inductive logic reasoning approach. First, audio recordings were transcribed verbatim. Thereafter, the first author (MM) read through the transcripts several times to have a detailed understanding of the data. Second, initial codes were made across the data. Thereafter, these codes were collated into initial themes. Fourth, initial themes were reviewed by examining possible connections between them. Afterwards, final themes were generated by bunching initial themes based on commonality. Finally, the most salient quotations were selected to represent final themes.

## Trustworthiness of the study

There are four criteria that should be considered by qualitative researchers in their pursuit of a trustworthy research study, namely credibility, transferability, dependability and confirmability (Lincoln and Guba, 1986). Efforts were also made to ensure that the study meets the trustworthy criteria.

Credibility addresses the extent to which findings are congruent with reality (Shenton, 2004). According to Lincoln and Guba (1986), one way of ensuring credibility is for researchers to ensure they are using well established research methods. In this research, phenomenological design was used. This is a tried and tested design in qualitative research methodology and has been used in many social research studies around the globe. In addition, as a way of confirming credibility, researchers are recommended to employ the process of iterative questioning. The interview guide’s length as well as the number of questions that probed the phenomenon of mandatory COVID-19 vaccination enabled the iterative nature of questioning. The questioning focused both on past and present in so far as the changing nature of professional work in Zimbabwe is concerned. It is also important to note that several debriefings were held between the two authors to come up with a sound interview guide which will measure what is should measure.

Transferability relates to external validity of a study. Since the findings of a qualitative project are specific to a small number of particular environments and individuals, it is impossible to demonstrate that the findings and conclusions are applicable to other situations and populations (Shenton, 2004). The notion of transferability has been a highly debatable issue in qualitative research. Several authorities argue that transferability is impossible owing to the fact that all observations are defined and are a product of the context in which they occur (Erlandson et al., 1993). It is, however, important to note that the key issue in qualitative research is not to search for the usual and traditional generalizability: rather, the objective is to seek an understanding and appreciation of the conditions under which a particular finding or phenomenon appears and operates (Lincoln and Guba, 1986). The sampling methods employed in this study are not representative. However, purposive sampling attempts to represent to an extent operations in Zimbabwe’s retail outlets regarding COVID-19 mandatory vaccination.

Dependability in qualitative studies corresponds to reliability. Reliability is addressed by employing techniques which show that if the work is to be repeated in the same context, using the same methods and with the same participants, similar results would be achieved. However, owing to the changing nature of issues addressed by qualitative researchers, such provisions of reliability are problematic in their work (Fidel, 1993; Marshall and Rossman, 2014). The dependability issue can be directly addressed by reporting the processes in the study in detail; by doing so, one would be enabling future researchers to repeat that particular study. Under such a scenario, the research design is viewed as prototype mode. In order to address the issue of dependability, the researchers devoted a section to describe the research methodology used in this study.

Relating to the notion of confirmability in qualitative research, scholars argue that it goes hand in hand with the aspect of objectivity (Patton, 2014). Objectivity in science is associated with the use of instruments that are independent of human skill and perception. However, real objectivity is difficult to achieve since tests and questionnaires are designed by human beings and so the researcher’s intrusion is inevitable (Patton, 2014).

Miles et al. (2018) noted that an important criterion for confirmability in qualitative research is the extent to which the investigator admits his/her own predispositions, agendas and assumptions. The researcher should declare and acknowledge his/her beliefs underpinning certain decisions made as well as methods adopted. Emphasis should also be on why the researcher favored a particular approach to research at the expense of other approaches. In this research, a section has been devoted to explain the qualitative approach which was deemed fit.

In addition, the researchers clearly highlighted their academic and professional background and confirmed that they have no interest in the way employees in the retail sector are managed.

Confirmability can also be ensured by the researcher’s ability to provide an “audit trail” (Shenton, 2004). This would allow an independent reader to trace the research step by step through the decisions made as well as procedures described. The researcher provided a detailed methodology section in an effort to address the notion of confirmability in this study.

## Findings and discussion

The findings are presented in three broad subsections. The focus is first on the extent to which employees have been consulted with regards to mandatory COVID-19 vaccination. The section continues to look into the perceptions of employees relating to mandatory COVID-19 vaccination. Ways through which employees are coping with mandatory COVID-19 vaccination marks the end of this section. Through continuous engagement with the data, inductive reasoning was used to discover themes. Table 2 below presents thee themes and sub-themes with each of these explained in detail in following sections:

**Table 2:** Themes and sub-themes of findings.

Theme	Sub-themes
The magnitude of consultation	Formal genuine consultations
	Formal less genuine consultations
	No consultation
Employees’ perceptions of COVID-19 mandatory vaccination	Perceived good decision
	Perceived tied hands of management
	Them and us perception
Coping with mandatory COVID-19 vaccination	Full vaccination
	Obtaining a vaccination card fraudulently
	Half-baked vaccination

## The magnitude of employee consultation

The study reveals that employees in the retails sector have been consulted on a continuum. The continuum ranges from high level consultation to non-consultation with regards to COVID-19 mandatory vaccination.

### Formal genuine consultation

It has emerged from the study that employees in the retail sector were rarely consulted on the issue of mandatory vaccination. Only two of the participants highlighted having been formally consulted. One participant, a 42-year-old make till operator had to say:

I was called by my manager to his office, and he asked me about the issue, and I gave my opinion on the matter. It was a good moment as I know that whatever the decision made, my voice was there as I had an opportunity to be heard.

In accordance with the arguments by Chu (2014), managers in the retail sector adopted an autocratic style of leadership. From these findings, it is clear that adequate formal consultations were not made in as far as mandatory COVID-19 vaccination. This is in contrast with the ideas of Bratton et al., 2021 who argue that contemporary organizations must seriously consider employee involvement as this yields positive results both for individuals and corporates. Findings of this study are also out of sync with the sentiments by Chanana (2021) who has it that management must make sure they involve employees in decisions made during a crisis. Above all, the approach taken by management is against the spirit of democratic management where participation of employees especially on issues that affect them is encouraged (Kilicoglu, 2018).

It is important to note that as highlighted by Du Plessis and Keyter (2020) and Ma and Yang (2020), particularly in the context of COVID-19, managers and employers could have had limited time to consult employees as this was a crisis. However, communication of the urgency in COVID-19 related issues mighty have helped employees understanding the predicament of managers. Furthermore, managers might have combined autocratic leadership style with some communications meant to demystify social media content that was circulating that led to COVID-19 vaccination resistance.

### Formal less genuine consultations

Although 2 of the participants indicated that they were formally consulted on mandatory COVID-19 vaccination, they argued that the consultations were mere window dressing efforts. Participants indicated that management had already made up their mind and the nature of consultation only required employees to rubber stamp a position. Participant 12, a 58-year-old male argued:

Yes, I was consulted on the issue of the mandatory covid-19 vaccination. However, they just brought the issue with an already existing position. They had already concluded the matter and all I had to do was to rubber stamp.

Participant 12 also indicated that while he was happy to be consulted and to have his view heard, he was not sure why management chose to consult him ahead of all other employees. He was not equally sure whether he was the only employee consulted or more other employees were consulted. In this regard, he had to say:

Up until now, I am not sure why management chose to consult me ahead of all other employees at this branch. On that note, I am not sure whether others were also consulted but I never heard anyone saying so. So, chances are high that they consulted me only.

In this case, it is clear that although management may argue that it has consulted some employees on the issue of mandatory COVID-19 vaccination, employees had a feeling that the COVID-19 vaccination was rather imposed on them. There was inadequate consultation. Employees after such perceived low-level consultation, may not feel as part and parcel of decision makers, especially on issues that concerns them. As predicted by Kilicoglu (2018), perceived use of an autocratic style of leadership may result in compromised commitment to decisions arrived at.

### No consultation

The majority of participants, indicated that they were not even consulted on the issue of COVID-19 mandatory vaccination. In this case, contrary to the position by Naqshbandi et al. (2018) advocating for employee consultation on issues that pertains them, managers in the retail sector in Zimbabwe did not consider it important to engage their employees on the issue of COVID-19 mandatory vaccination.

Possible direct and indirect reasons can be cited for management’s position of failing to consult employees. First and foremost, it is highly likely that management took advantage of prevailing economic environment obtaining in Zimbabwe where demand for employment exceeds its supply. In such a situation, management could have been fully aware that employees were unlikely to leave the organization and they were also highly likely to comply with the directive to get vaccinated. Second, management, In line with the argument by Kougiannou et al. (2021) who indicated that there are moments when management should make quick decisions. Health related aspects such as those relating to COVID-19 require quick decisions which may render major employee consultations time consuming. The need to protect other stakeholders such as customers and suppliers. This might have compounded management not to consult employees as they had to make a quick decision. However, even though managers had inadequate time and as argued by Du Plessis and Keyter (2020) and Ma and Yang (2020), crisis moments require quick decisions that may not allow adequate consultations, related communication regarding COVID-19 and its associated risks should have been done.

On the other hand, management might have just felt that it is their right and prerogative to make important decisions on behalf of their employees (Hodgkinson, 2018; Kougiannou et al., 2021). This is against the contemporary spirit of employee engagement and has serious reparations (García et al., 2018; Bratton et al., 2021).

Although COVID-19 presented a crisis to organizations and called for management to act in accordance with the autocratic management style, there was need to compliment this approach with some aspects of democratic management. Furthermore, management might have considered combining autocratic management style with formal engagements with employees meant to justify their position at the same time, demystifying vaccination myths and misunderstandings which were circulating mainly on social media platforms.

## Employees’ perceptions of COVID-19 mandatory vaccination

The study has established different perceptions of COVID-19 vaccinations by retail employees. Generally, these perceptions revealed satisfaction to dissatisfaction as outlined in the following sections.

### Perceived good decision

Four participants highlighted that management of the retail sector took a good decision by forcing employees to undergo a forced vaccination exercise. Participant 18, a 32 year-old female had this to say:

Forced vaccination was good as it was meant to save the life of everyone in and outside an organization. Given the nature of the pandemic, had I been holding a management position, I would have done the same.

The findings of this study resonates with the work of Bridoux and Vishwanathan (2020) who argue that management decisions must be in the interest of all the stakeholders of an organization, in this case, stakeholders of a retail outlet. The stakeholders include employees, customers, and suppliers. One participant, although he was in support of the move by management, he argued that management should have consulted employees as a major stakeholder as outlined below by a 27-year-old male participant.

The idea of forced vaccination was good; however, management should have consulted employees. They should have made joint decisions together with us. I just wonder why they did not engage us. Maybe they see us as dull and unable to understand the risk associated with COVID-19.

The above quotation clearly indicates that although some retail employees agreed with the decision of mandatory vaccination, they were not happy for not being consulted on the issue. In this context, some employees were satisfied with the decision made regarding mandatory COVID-19 vaccination but were dissatisfied with the decision made by management of not engaging them. Literature (García et al., 2018; Dahmardeh and Nastiezaie, 2019; Bratton et al., 2021) has it that when involved in decision making, employees are happy and are committed to the decision made. The exclusion of employees from decision making, especially on issues that pertains them may result in disgruntlement and resistance to organizational initiatives.

### Perceived tied hands of management

Another theme which emerged from the study was that of management having no option but to proceed and make it mandatory for employees to undergo a mandatory vaccination process for them to continue rendering their services to the employer. Five of the participants highlighted that although they were not consulted on the issue of mandatory COVID-19 vaccination, management had no option but to proceed given the severity of the pandemic. Participant 14 a 33-year-old male argued as follows:

Covid-19 has been very ruthless and because of this, management had no option but to quickly decide. I am sure there was no time for consultations. Management would have been naive to consult employees whilst covid-19 was ravaging day in and day out.

Unlike participants in the previous section, these ones were comfortable with the decision by management regarding COVID-19 and understood the position of management of not consulting employees given the seriousness of the pandemic. Perceptually, these participants exonerated management for not consulting them. The fact that these participants acknowledged the dilemma faced by management meant that chances of the former being up in arms with the latter were minimal.

### Them and us perception

The majority of participants 13 argued that management did not consult them because they consider themselves superior to the employees. In this context, participating employees were not happy not being given a chance to contribute to the decision on COVID-19. According to them, the decision by management not to consult them emanated not from the severity of the pandemic, and not from the limited time management had to make a decision, but from the fact that management considered themselves superior to employees. Management considered themselves as more intelligent and in a class of their own opposed to employees who were seen as inferior and unable to make sound decisions. Participant 7, a 26-year-old female employee had this to say with regards to mandatory COVID-19 vaccination’

They are the managers, they are the decision makers. They say, and we do. No one can question them…their word is final. They are convinced we are dull and they are clever. All they want us to do is to get vaccinated even against our will. Our choices are limited.

The tone and choice of words by the above participant clearly brings out a strong level of dissatisfaction against being excluded from making a decision on an issue that directly affect employees. The findings of this study pertaining to the perceived exclusion of employees and their subsequent feelings concur with the arguments of some scholars (García et al., 2018; Dahmardeh and Nastiezaie, 2019; Bratton et al., 2021) who have it that workers gets disgruntled when they are not involved in decision making processes, especially on issues that concern them.

Findings clearly reveal the concept of othering at play as perceived by some participants. Othering entails an out-group and an in-group where perceived differences do not allow the 2 groups to formally meet and discuss issues at the workplace. Inadequate or lack of formal interactions between management and employees have been argued to cause the former to have a prerogative to make unilateral decisions even on issues that affect employees directly.

It is evident from the findings that employees can easily recognize the demarcation between them and the managers leading to the latter having power over the former. Sentiments from most participants clearly reveals their disgruntlement of not being consulted, particularly on issues that pertains them. The power and importance of a democratic leadership style was emphasized (Kilicoglu, 2018).

## Coping with mandatory COVID-19 vaccination

It has emerged from the study that employees reacted differently to the mandatory COVID-19 vaccination. To cope with the mandatory COVID-19 vaccination, some participants complied with the order, regardless of whether they wanted to or not. Some settled to just fraudulently obtain a vaccination card, while some just went for a first dose and never returned for a second dose. These reactions are explained below.

### Full vaccination

Five of the participants admitted having paid heed to the call by management for mandatory COVID-19 vaccination. These participants reported to have gone through both doses of the vaccination. Findings further reveal that 2 of these were convinced that this was a good idea and were happy to get vaccinated. As alluded to by participant 13, a 33-year-old employee the only way to fight the pandemic was to get vaccinated. He had to say:

Look at how people have been dying…it is so pathetic. Vaccinations have always proved to be effective since we were young. We previously had a pandemics such as polio and measles, but were conquered by vaccinations. At least as of now, that is what we have at our disposal as we navigate into the future.

This could have resulted from the massive campaigns by the government and other not for profit organizations on mass media platforms encouraging Zimbabweans to get vaccinated in order to contain the pandemic.

Although the remaining three participants also went for full vaccination, they felt they had no option because their respective organizations had made it mandatory. As indicated by participant 19, a 44-year-old male employee, the move by his organization was coercive; in this regard, he argued:

What was I supposed to do? Failure to comply with the directive meant that I would lose employment. Honestly, I did not want this, but I had no option except to be vaccinated.

The prevailing economic condition in Zimbabwe characterized by an excess supply of labor might have contributed to some participants opting to get vaccinated even when they did not want to. As alluded to by Mudzonga (2021), the balance of power in the workplace has tilted in favor of the employer and against the employee who can easily be replaced in Zimbabwe. Although the five were all fully vaccinated, their motivations to do so was different with others out of conviction and others out of fear of losing their employment.

### Obtaining a vaccination card fraudulently

Findings have it that some employees connived with some health personnel to obtain vaccination cards using unorthodox means. This category composed of 9 (45) of the participants. Participant 16, a 36-year-old employee shared his experiences regarding an ill-gotten vaccination card:

A friend’s girlfriend is a nurse…we approached her for some [vaccination] cards with all the details. She provided these cards at a cost citing that it is a syndicate and she shares the money with her superiors. The syndicate wanted the money, and we wanted the vaccination cards.

Obviously, this category of employees did not want to be vaccinated at the same time, they did not want to lose their employment. They then met health personnel who were eager to earn extra money through corrupt behaviors. Corruption in the acquisition of COVID-19 cards not only in Zimbabwe, but in other countries has also been noted by other scholars (Maketo and Mutizwa, 2021; Tshabangu and Salawu, 2021; Sorooshian et al., 2022).

It is possible that had management earnestly and fully consulted employees on the need to get vaccinated, the latter would have expressed their fears and management together with other stakeholders would have collectively worked on that to make sure the fears are dealt with. The majority of employees had fears derived from some unfounded arguments which circulated on social media platforms. In line with this, Dzinamarira et al. (2021) have it that COVID-19 vaccination was negatively seen in some social and religious circles, thereby leading to some sectors resisting it.

In this case, findings show that participants had inadequate knowledge regarding COVID-19 vaccination as they were not [adequately] consulted. For commitment to organizational decisions, employees must be involved in the process as managers exercise democratic leadership style (Kilicoglu, 2018). Although the decision for mandatory COVID-19 might have been perceived to be autocratic, providing formal communication pertaining to COVID-19 vaccination could have gone a long way in preparing employees for vaccinations on a rather willing basis.

### Half-baked vaccination

It has been revealed from the findings of this study that some participants only settled for the first dose and were reluctant to go for the second dose. Six of the participants constituted this category. The idea, as highlighted by the majority in this category was to lie somewhere in the middle, having started the journey, but not bringing it to completion. In line with this reaction to mandatory COVID-19 vaccination, participant 17, a 24-year-old male employee presented his case as follows:

We were afraid to lose our employment, so my close friends and I agreed that we go for the first dose, then wait and see. We presented to the human resource department a card with a single dose and they filed it. Since then, nothing has been said regarding the second dose and as of now, we are happy that way. We will consider the second dose the moment they start making noise about it, but as of now, they are quiet.

The above quote clearly reveals that some people responded to the mandatory COVID-19 vaccination out of fear of losing employment more than the fear of getting infected with the pandemic.

As previously highlighted and in line with autocratic leadership style, Chu (2014), employees are naturally reluctant to commit to decisions they would not have participated in. In most cases, if they decide to follow the directives, it would be out of fear of possible punishment from management. Autocratic leadership thrives on instilling fear of punishment (Chu, 2014; Erdem, 2021). Having included some tenets of democratic leadership in their approach to mandatory COVID-19 could have helped employees unpack the dynamics of the pandemic and made informed decisions.

## Conclusion and recommendations

The study findings reveal that although organizations in the retail industry made it mandatory for their employees to get COVID-19 vaccination, the decision was made in the absence of employees. The exclusion of employees by management was largely interpreted by the former as unfair and unnecessary given the fact that they were going to be directly affected by the decision. It has emerged from the study that most retail workers saw themselves being at the mercy of their managers with the latter making unilateral decisions, even on matters that pertains to employees. Employees generally reacted to the exclusion by engaging in corrupt acquisition of the COVID-19 vaccination card as well as going for a single dose as they adopted a watch and see attitude. Employees revealed tendencies of indirectly defying the mandatory COVID-19 vaccination directive by their respective organizations but could not directly air out their views because they feared losing employment.

As recommendations, even during a crisis, organizations must thrive to include employees in making decisions, particularly on issues that pertain the latter. Democratization of the workplace has been found to go a long way in making employees comply with the organization’s resolutions, even in times of a crisis. This can be achieved through making use works councils or internally organized surveys. In life threatening situations, such as COVID-19, management may consider conducting some workshops to clarify issues. Future studies may benefit from engaging managers on the extent to which employees are consulted and the platforms as well as the strategies used.

Future studies may consider taking on board other sections of the economy such as SMEs, institutions of learning, transport among others. Different sectors may reveal different perceptions. It could also be interesting to investigate perceptions of mandatory COVID-19 vaccination of professionals such as lawyers, medical doctors among others.

## Contributions of the study

Unlike previous studies on COVID-19 in Zimbabwe (Chigevenga, 2020; Mbunge et al., 2020; Murewanhema and Makurumidze, 2020; Mackworth-Young et al., 2021) which attended to such issues as responses toward the anticipated vaccines, role of emerging technologies, health service delivery and community perspectives, this study focused on the perceptions of retail sector employees on mandatory vaccination from an autocratic-democratic perspective by management. In addition, the study contributed by linking the perceived level of consultation to their responses to mandatory COVID-19 vaccination. Furthermore, looked at the perceptions of mandatory COVID-19 vaccinations from an economically devastated country where unemployment rate is high.

## Limitations of the study

Interviews were conducted and employers premises nowadays due to technology they might be cameras hence employees could have left out some important information and in most cases they were conducted during working hours hence employees were in a hurry to respond to questions. It is possible that some important information could have been left out by participants due to limited time. In addition, these employees might have felt restrained since the study was conducted by strangers.

## Author’s note

The pandemic COVID-19 brought a number of uncertainties to the organizations and these organizations introduced mandatory COVID-19 vaccination to curb the spread of the pandemic. This paper therefore helps to bring the reactions and perceptions of employees to mandatory COVID-19 vaccination using a qualitative approach in a precarious environment.

## Data availability statement

The raw data supporting the conclusions of this article will be made available by the authors, without undue reservation.

## Ethics statement

Ethical review and approval was not required for the study on human participants in accordance with the local legislation and institutional requirements. The patients/participants provided their written informed consent to participate in this study.

## Author contributions

All authors listed have made a substantial, direct, and intellectual contribution to the work and approved it for publication.

## Conflict of interest

The authors declare that the research was conducted in the absence of any commercial or financial relationships that could be construed as a potential conflict of interest.

## Publisher’s note

All claims expressed in this article are solely those of the authors and do not necessarily represent those of their affiliated organizations, or those of the publisher, the editors and the reviewers. Any product that may be evaluated in this article, or claim that may be made by its manufacturer, is not guaranteed or endorsed by the publisher.
